# Supplementary material for: The catalytic mechanism of the RNA methyltransferase METTL3
Source: eLife. 2024 Mar 12;12:RP92537. doi: 10.7554/eLife.92537 (PMC10932547; doi:10.7554/eLife.92537)
Supplement: Supplementary file 1. — Statistics for the highest-resolution shell are shown in parentheses. [file elife-92537-supp1.docx]

|  | BA1 complex  (PDB 8PW9) | BA2 complex  (PDB 8PW8) | BA4 complex  (PDB 8PWA) | BA6 complex  (PDB 8PWB) |
| --- | --- | --- | --- | --- |
| **Data collection** | | | | |
| Wavelength (Å) | 1 | 1 | 1 | 1 |
| Resolution range (Å) | 49.69 - 2.301  (2.383 - 2.301) | 44.51 - 2.3  (2.382 - 2.3) | 44.54 - 2.1  (2.175 - 2.1) | 49.52 - 2.5  (2.589 - 2.5) |
| Space group | P 32 2 1 | P 32 2 1 | P 32 2 1 | P 32 2 1 |
| Unit cell (Å, °) | 63.94 63.94 225.14,  90 90 120 | 63.78 63.78 225.45,  90 90 120 | 63.89 63.89 225.18,  90 90 120 | 63.69 63.69 224.77,  90 90 120 |
| Total reflections | 237414 (22942) | 218643 (19326) | 311563 (28191) | 182166 (17587) |
| Unique reflections | 24644 (2396) | 24575 (2365) | 32185 (3137) | 19170 (1859) |
| Multiplicity | 9.6 (9.6) | 8.9 (8.1) | 9.7 (8.9) | 9.5 (9.4) |
| Completeness (%) | 99.80 (99.30) | 99.72 (99.41) | 99.72 (99.08) | 99.78 (99.79) |
| Mean I/sigma(I) | 17.40 (1.82) | 15.83 (1.61) | 18.28 (1.50) | 15.90 (1.75) |
| Wilson B-factor | 45.64 | 43.17 | 38.9 | 49.72 |
| R-merge | 0.101 (1.204) | 0.1072 (1.267) | 0.0969 (1.317) | 0.1241 (1.244) |
| R-meas | 0.1068 (1.272) | 0.1138 (1.351) | 0.1024 (1.398) | 0.1313 (1.315) |
| R-pim | 0.03422 (0.4072) | 0.0376 (0.456) | 0.03266 (0.4639) | 0.04229 (0.4233) |
| CC1/2 | 0.999 (0.644) | 0.999 (0.6) | 0.999 (0.61) | 0.998 (0.627) |
| CC* | 1 (0.885) | 1 (0.866) | 1 (0.87) | 1 (0.878) |
| **Refinement** | | | | |
| Reflections used in refinement | 24616 (2396) | 24523 (2365) | 32118 (3137) | 19134 (1859) |
| Reflections used for R-free | 1230 (120) | 1229 (119) | 1606 (157) | 958 (93) |
| R-work | 0.1921 (0.2542) | 0.1971 (0.2973) | 0.1907 (0.2731) | 0.1999 (0.3001) |
| R-free | 0.2383 (0.3146) | 0.2487 (0.3502) | 0.2250 (0.3064) | 0.2450 (0.3605) |
| CC(work) | 0.957 (0.834) | 0.956 (0.774) | 0.963 (0.814) | 0.954 (0.758) |
| CC(free) | 0.943 (0.675) | 0.949 (0.706) | 0.937 (0.809) | 0.937 (0.675) |
| Number of non-hydrogen atoms | 3648 | 3621 | 3742 | 3518 |
| macromolecules | 3473 | 3437 | 3480 | 3416 |
| ligands | 45 | 51 | 44 | 42 |
| solvent | 130 | 133 | 218 | 60 |
| Protein residues | 442 | 437 | 440 | 433 |
| RMS bonds (Å) | 0.019 | 0.018 | 0.018 | 0.019 |
| RMS angles (°) | 1.1 | 1.12 | 1.07 | 1.12 |
| Ramachandran favoured (%) | 96.73 | 96.91 | 97.89 | 95.68 |
| Ramachandran allowed (%) | 3.04 | 2.38 | 1.64 | 4.32 |
| Ramachandran outliers (%) | 0.23 | 0.71 | 0.47 | 0 |
| Rotamer outliers (%) | 0.28 | 0.28 | 0.55 | 1.69 |
| Clashscore | 3.38 | 3.85 | 3.65 | 6.71 |
| Average B-factor | 49.58 | 45.56 | 43.61 | 51.29 |
| macromolecules | 49.15 | 45.36 | 43.28 | 51.08 |
| ligands | 86.54 | 61.52 | 54.49 | 66.93 |
| solvent | 48.18 | 44.49 | 46.55 | 52.27 |
